# Effect of *Nigella sativa* fixed and essential oils on antioxidant status, hepatic enzymes, and immunity in streptozotocin induced diabetes mellitus

**DOI:** 10.1186/1472-6882-14-193

**Published:** 2014-06-17

**Authors:** Muhammad Tauseef Sultan, Masood Sadiq Butt, Roselina Karim, Shahzad Zafar Iqbal, Shakeel Ahmad, Muhammad Zia-Ul-Haq, Luigi Aliberti, Atif Nisar Ahmad, Vincenzo De Feo

**Affiliations:** 1 Faculty of Food Science and Technology, University of Putra Malaysia, Putra, Malaysia; 2 Department of Food Sciences, Bahauddin Zakariya University Multan, Multan, Malaysia; 3 National Institute of Food Science & Technology, University of Agriculture, Faisalabad, Pakistan; 4 Department of Agronomy, Bahauddin Zakariya University Multan, Multan, Pakistan; 5 The Patent Office, Karachi, Pakistan; 6 Department of Pharmacy, University of Salerno, Salerno, Italy; 7 Department of Pathobiology, Bahauddin Zakariya University Multan, Multan, Pakistan

**Keywords:** *Nigella sativa*, Traditional uses, Diabetes mellitus, Hyperglycemia, Oxidative stress

## Abstract

**Background:**

Nigella sativa fixed (NSFO) and essential (NSEO) oils have been used to treat diabetes mellitus and its complications. Present study was undertaken to explore and validate these folkloric uses.

**Methods:**

Sprague dawley rats having streptozotocin (STZ) induced diabetes mellitus were used to assess the role of NSFO and NSEO in the management of diabetes complications. Parameters investigated were antioxidant potential, oxidative stress, and the immunity by *in vivo* experiments.

**Results:**

The results indicated that STZ decreased the glutathione contents (25.72%), while NSFO and NSEO increased the trait significantly (P < 0.05). Experimental diets increased the tocopherol contents (P < 0.01) and enhanced the expression of hepatic enzymes (P < 0.01). Correlation matrix further indicated that antioxidant potential is positively associated (P < 0.05) responsible for the modulation of hepatic enzymes and the decrease of the nitric oxide production thus controlling the diabetes complications.

**Conclusions:**

Overall, results of present study supported the traditional use of *N. sativa* and its derived products as a treatment for hyperglycemia and allied abnormalities. Moreover, *N. sativa* fixed and essential oils significantly ameliorate free radicals and improve antioxidant capacity thus reducing the risk of diabetic complications.

## Background

Diabetes mellitus is a matter of great concern for the medical and allied stakeholders. According to an estimate, at the end of year 2030, approximately 376 million peoples will be the victims of diabetes mellitus [1]. The hyperglycemia, decreased insulin production/insulin sensitivity along with some lifestyle related problems including poor dietary habits, and hypertensions are major causes of diabetes mellitus and its complications [2]. Diabetes mellitus is associated with enhanced production of free radicals that further complicate the situation resulting in oxidative stress, cardiovascular disorders, renal failure, neurodegeneration, and immune dysfunction [3].

In diabetes mellitus, the human defence system responds slowly and the production of free radical tends to increase due the decreased scavenging abilities of the body [4,5]. Diabetes induced oxidative stress needs to manage properly in clinical practices. In order to regulate free radical production, nature bestowed humans with complex defence mechanism comprising of different organ, enzymes, chemicals, and mediators. The system is effective in scavenging free radicals but diabetes mellitus can result in imbalance that can lead to DNA damage, myocardial infraction, LDL oxidation, and inflammation [6]. The nutritional status of diet has significant impact on defence system of the body but deficiency of certain antioxidants/nutrients enhances the complications. Consumption of antioxidant rich foods may improve antioxidant defence mechanism and provide protection against oxidative damage caused by free radicals [7].

Many traditional plants products are in use since long due to their therapeutic potential e.g. garlic, green tea, ginger etc., and several avenues are yet to be explored [6]. *Nigella sativa* L. belongs to family *Ranunculaceae* and its different parts of plant are in use for medicinal purposes to cure various maladies [8]. The available literature reports the plant for its antioxidant activity due to presence of bioactive molecules that are mainly concentrated in fixed or essential oil including tocopherols, phytosterols, polyunsaturated fatty acids, thymoquinone, ρ-cymene, carvacrol, t-anethole and 4-terpineol [9]. *N. sativa* has been utilized in some traditional medicines due to its hypothetical perceived antidiabetic properties. Some research studies have enumerated its ability to ameliorate oxidative stress and nephrotoxicity [10]. Some other studies also reported its insulinotropic properties and ability to maintain β-cells integrity along with effectiveness in lowering cholesterol and drug toxicity [11]. *N. sativa* normalizes the level of hepatic enzymes in normal rat, e.g. γ-glutamyl transpeptidase, xanthine oxidase, blood urea nitrogen, serum creatinine and extent of lipid peroxidation [12,13]. However, the effects of *N. sativa* on oxidative stress in diabetes mellitus need further clarification.

For the purpose, present research explored the role of *N. sativa* fixed and essential oil against diabetes induced oxidative stress. The antioxidant status was determined by measuring the serum tocopherol and glutathione contents. Moreover, by using liver tissue homogenate, the level of hepatic enzymes were assayed e.g. sodium dismutase (SOD), catalase (CAT), glutathione reductase (GR), superoxide dismutase (SOD), glutathione peroxidase (GPx) and catalase (CAT), and myeloperoxidase (MPO). The impact of supplementation of NSFO and NSEO on nitric oxide and xanthine oxidase was also assessed. Multiple correlations, interdependence of these parameters on each were also reported.

## Methods

### Collection of Nigella sativa and extraction of fixed and essential oils

The Barani Agricultural Research Institute, Chakwal, Pakistan provided us with *N. sativa* seeds (Voucher/Specimen No. Chk.Pk-926). Chemical Reagents (analytical & HPLC grade) and standards were purchased from Sigma-Aldrich Tokyo, Japan and Merck KGaA, Darmstadt, Germany. National Institute of Health (NIH) Islamabad, Pakistan provided infectious free Sprague Dawley rats for the research purpose as per instructions of “Animal Care Committee, NIFSAT-Faisalabad Pakistan”. The seeds of *N. sativa* were slurred with hexane (in the ratio of 1:6 using a Soxhlet apparatus and later solvent was removed using rotary evaporator) to extract the fixed oil. *N. sativa* essential oil was extracted using locally assembled hydro-distillation apparatus.

### Housing of rats

The National Institute of Health (NIH), Islamabad provided infection-free 30 Sprague Dawley rats that were further divided into three groups of ten rats each. The animals were maintained according to standard guidelines of Animal Institute of Nutrition (AIN), USA i.e. temperature 23 ± 2°C, relative humidity 55 ± 5%, and 12-hr light–dark cycle. In the first week, the feed of the rats was basal diet in order to acclimatize them to new environment. Later, rats received their respective experimental diets (Table 1) for a period of eight weeks (56 days). At 28 and 56 days of feeding trials, five rats from each group were decapitated for blood collection through neck and cardiac puncture [14]. The collected blood samples were analyzed for further assays and details are mentioned herein.

**Table 1:** Diet plan used in the study

**Groups**	**Diets**
**I**	D_1_: (Control/placebo diet)
**II**	D_2_: (4.0% fixed oil)
**III**	D_3_: (0.3% Essential oil)

### Antioxidant status

Glutathione contents were determined following the protocols described by Beutler [15]. The product of GSH + DTNB in the protein free supernatant was measured using spectrophotometer at 412 nm (expressed as nmol/mg protein). Level of serum α-tocopherol and γ-tocopherol were also measured following the procedures described by Xu and Godber [16] through HPLC. Briefly, the oil samples were slurred in hexane in the first step until dissolved completely. A normal phase HPLC column (250 mm × 4.6 mm, 5.0 μm particle size) and mobile phase consisting of isooctane/ethyl acetate (96:4 v/v) was used for the purpose. Total run time and flow rate were 30 min and 1.0 mL/min, respectively. The detector was set at 290 nm excitation wavelength and 400 nm emission wavelengths. The column temperature was 35°C. Similarly, the individual standards of isomers of tocopherols were run using the same pattern and curves were obtained and used for calibration in order to determine the amounts of tocopherols in *N. sativa* fixed oil.

### Hepatic antioxidant enzymes assays

The estimation of superoxide dismutase (SOD) activity requires measuring the ability of enzyme to inhibit cytochrome ‘c’ oxidation [17] and activity was expressed in IU/mg protein. The decomposition of hydrogen peroxide was measurement of by-products was mainly used for the estimation of catalase (CAT) activity and the units remained the same as IU/mg protein and one IU was equivalent to one μmol H_2_O_2_ consumed per mg protein per minute [18]. Glutathione transferase (GST) action was measure using the commercial kits provided by Bioassay Internationals. The reaction include the rate of formation of conjugate between GSH and 1-chloro-2,4- dinitrobenzene [15] and measurement unit used IU/ mg protein. One IU is equivalent to one μmol of conjugate formed/min/mg protein. Glutathione peroxidase (GPX) activity was estimated using tertiary butyl hydroperoxide (tbHP) as substrate [19] and the activity was expressed in IU/mg protein and one IU equivalent to one nmol of NADPH oxidized/mg protein in one minutes. Glutathione reductase (GR) activity was assayed by following the oxidation of NADPH. Glutathione reductase (GR) activity was assayed at 37°C and 340 nm by following the oxidation of NADPH. Hepatic enzymes like GST, GPx and GR was determined as described by Paglia and Valentine [20].

### Myeloperoxidase and xanthine oxidase activity

Using spectrophotometer, we estimated the activity of tissue associated myeloperoxidase (MPO) following the procedures laid down by Hillegas *et al.*[21]. For the purpose, a single unit of enzyme activity (IU/mg of proteins) was defined as the amount of the MPO present that caused a change in absorbance measured at 460 nm during the reaction time of three minutes. The xanthine oxidase was measured using the using diagnostic kits from Cayman Chemicals according following the procedures mentioned by Marcocci *et al.*[22].

### Statistical analysis

Statistical package i.e. Cohort V-6.1 (Co-Stat Statistical Software, 2003) was used for data analysis. The values presented in Tables are means ± standard deviation. The technique of analysis of variance (ANOVA) was applied to check the level of significance. The source of variations (SOV) were diets (factor A), intervals (factor B) and their interaction (A × B). Duncan’s multiple range test (DMRt) further clarified the effects of diets in a comprehensive manner.

## Results

The process of oxidation is an integral part of energy yielding metabolism. However, the same process is responsible for the production of free radicals/reactive oxygen species (ROS) that can be destructive for human health [4]. In some pathological states like diabetes mellitus, the onset of oxidative stress is very common [5], and it need to be mediated through integrated approach of boosting natural defence through natural antioxidant [3]. In the present research, we observed the protective effects of *N. sativa* fixed and essential oil against oxidative stress with special reference to antioxidant status and hepatic enzymes along with their role as enhancing the immunity.

### Indices of antioxidant status

**Figure 1:**
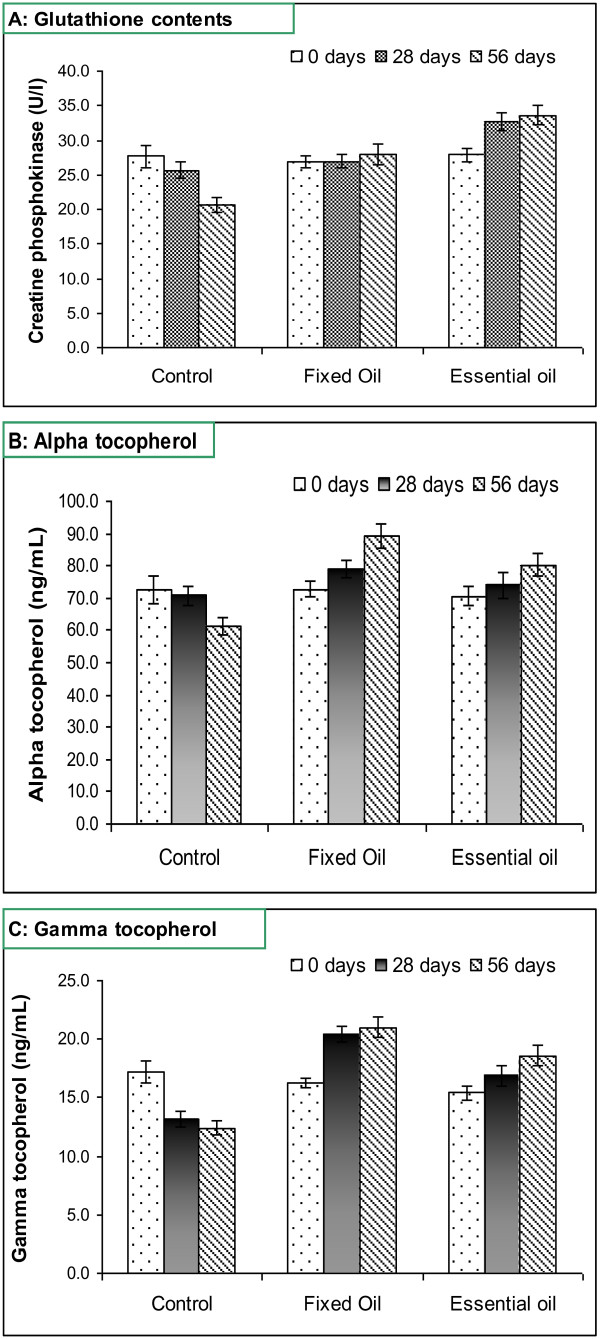
**Antioxidant potential of *Nigella sativa *fixed and essential oils. (A)** represents glutathione contents and **(B)** and **(C)** show the α- and γ- tocopherol contents. The antioxidant potential increased with the supplementation of fixed and essential oils. The essential oil increased glutathione contents, whilst fixed oil increased significantly tocopherol content.

The results regarding antioxidant status (Figure 1) explicated that diets and interaction affected glutathione, α-tocopherol, and γ-tocopherol contents significantly. The maximum glutathione (31.39 ± 1.79 mg/dL) was observed in *NSEO* group followed by fixed oil group (27.29 ± 0.34 mg/L) while the minimum value (24.63 ± 2.12 mg/L) was recorded for a control. During the course of study, marked decrease in glutathione content from 27.68 ± 1.60 to 20.56 ± 1.04 mg/L was observed in control whilst an increase from 26.93 ± 1.13 to 27.97 ± 1.23 and 27.85 ± 1.06 to 33.65 ± 1.49 mg/L, was recorded, respectively for NSFO and NSEO groups. Likewise, control group showed least α- and γ-tocopherols contents that decreased from 76.61 ± 4.42 to 61.16 ± 3.10 ng/mL and 17.19 ± 0.99 to 12.42 ± 0.63 ng/mL, respectively, during the 56 days study. Experimental diets containing NSFO and NSEO showed progressive improvements for both these traits i.e. α-tocopherol increased from 72.78 ± 2.77 to 89.27 ± 3.96 and 16.28 ± 0.68 to 21.03 ± 0.93 ng/mL, respectively, whilst γ-tocopherol from 70.67 ± 2.96 to 80.32 ± 3.54 and 15.42 ± 0.59 to 22.60 ± 1.00 ng/mL, respectively.

### Hepatic antioxidant enzymes

It is obvious from means pertaining to SOD (Table 2) that NSFO and NSEO decreased SOD contents significantly from 17.27 ± 0.722 to 16.90 ± 0.744 IU/mg protein and 18.11 ± 0.690 to 12.71 ± 0.564 IU/mg protein, respectively. In contrary, a significant increase in SOD contents was observed in control from 18.64 ± 1.076 to 22.43 ± 1.137 IU/mg protein. Likewise, catalase activity increased in control group from 8.57 ± 0.326 to 12.71 ± 0.564 IU/mg protein while in NSFO and NSEO groups, activity decreased significantly from 9.59 ± 0.401 to 8.49 ± 0.374 and 8.63 ± 0.498 to 7.94 ± 0.403 IU/mg protein, respectively.

**Table 2:** **Effects of *N. sativa *** **fixed and essential oil on sodium dismutase & catalase in diabetic rats**

**Parameters**	**Diets**	**Study intervals (Days)**		
		**0**	**28**	**56**
**SOD (IU/mg protein)**	**D**_**1**_	18.64 ± 1.076c	19.65 ± 0.637b	22.43 ± 1.137a
	**D**_**2**_	17.27 ± 0.722de	18.92 ± 0.636c	16.90 ± 0.744e
	**D**_**3**_	18.11 ± 0.690 cd	13.65 ± 0.665f	12.71 ± 0.564f
**Catalase (IU/mg protein)**	**D**_**1**_	8.57 ± 0.326d	9.99 ± 0.486b	12.71 ± 0.564a
	**D**_**2**_	9.59 ± 0.401b	9.20 ± 0.309c	8.49 ± 0.374d
	**D**_**3**_	8.63 ± 0.498d	8.62 ± 0.279d	7.94 ± 0.403e

Means sharing same letters in a column/row do not differ significantly at P < 0.05.

D_1_ = control diet; D_2_ *= N. sativa* seed fixed oil; D_3_ = *N. sativa* seed essential oil.

The streptozotocin decreased glutathione peroxidase activity significantly as witnessed in control from 65.68 ± 3.790 to 58.81 ± 2.981 IU/mg protein (Table 3), whereas the activity of same trait increased in experimental diets. Likewise, glutathione reductase increased in NSFO and NSEO groups from 15.53 ± 0.649 to 23.80 ± 1.048 and 15.60 ± 0.594 to 29.36 ± 1.303 IU/mg protein, respectively, whereas decreased in control group from 16.28 ± 0.939 to 14.60 ± 0.740 IU/mg protein. It is apparent from Table 3 that glutathione transferase exhibited a progressive increase while decreased in control group.

**Table 3:** **Effects of *N. sativa *** **fixed and essential oil on hepatic antioxidants enzymes in diabetic rats**

**Parameters**	**Diets**	**Study intervals (Days)**		
		**0**	**28**	**56**
**Glutathione peroxidase (IU/mg protein)**	**D**_**1**_	65.68 ± 3.790d	62.50 ± 2.025de	58.81 ± 2.981e
	**D**_**2**_	62.50 ± 2.615de	72.20 ± 2.427c	78.70 ± 3.466b
	**D**_**3**_	62.86 ± 2.394d	77.75 ± 3.785b	96.96 ± 4.304a
**Glutathione reductase (IU/mg protein)**	**D**_**1**_	16.28 ± 0.939d	15.53 ± 0.503de	14.60 ± 0.740e
	**D**_**2**_	15.53 ± 0.649de	18.33 ± 0.616c	23.80 ± 1.048b
	**D**_**3**_	15.60 ± 0.594de	24.35 ± 1.185b	29.36 ± 1.303a
**Glutathione transferase (IU/mg protein)**	**D**_**1**_	0.75 ± 0.043d	0.74 ± 0.024d	0.65 ± 0.033e
	**D**_**2**_	0.74 ± 0.031d	0.84 ± 0.028c	0.95 ± 0.042b
	**D**_**3**_	0.73 ± 0.028d	0.80 ± 0.039c	1.03 ± 0.046a

Means sharing same letters in a column/row do not differ significantly at P < 0.05.

D_1_ = control diet; D_2_ *= N. sativa* seed fixed oil; D_3_ = *N. sativa* seed essential oil.

### Immunopotentiating activity

Diets, study intervals, and their interaction showed significant effect on myeloperoxidase (MPO), xanthine oxidase and nitric oxide level with the exception of non-momentous effect of study intervals on nitric oxide. The streptozotocin injection enhanced the expression of myeloperoxidase (MPO) that differed significantly in various diet groups. The maximum activity (17.88 ± 1.466 IU/mg protein) was recorded in control, while NSFO and NSEO groups were statistically at par with less activity as compared to control (Table 4). Likewise, the maximum activity of xanthine oxidase in control group (29.36 ± 1.75 IU/mg protein) followed by fixed oil group (27.47 ± 1.20 IU/mg protein) whereas the minimum activity (25.04 ± 2.99 IU/mg protein) was observed in essential oil group. During eight-week study, xanthine oxidase activity decreased significantly from 29.03 ± 1.21 to 25.10 ± 1.11 and 29.45 ± 1.12 to 19.33 ± 0.86 IU/mg protein in NSFO and NSEO groups, respectively, and increased in control group. Means regarding nitric oxide (Table 4) demonstrated that group of rats fed with control diet showed a progressive increase from 23.42 ± 1.35 to 27.13 ± 1.38 nmol/dL, whereas fixed oil group reduced the level from 23.47 ± 0.98 to 21.22 ± 0.93 nmol/dL. *N. sativa* essential oil treatment appeared more potent in modulating nitric oxide production.

**Table 4:** **Effects of *N. sativa *** **fixed and essential oil on indices of immune system in diabetic rats**

**Parameters**	**Diets**	**Study intervals (Days)**		
		**0**	**28**	**56**
**Xanthine oxidase (IU/mg protein)**	**D**_**1**_	26.13 ± 1.51c	29.82 ± 0.97b	32.13 ± 1.63a
	**D**_**2**_	29.03 ± 1.21b	28.28 ± 0.95bc	25.10 ± 1.11d
	**D**_**3**_	29.45 ± 1.12b	26.33 ± 1.28 cd	19.33 ± 0.86e
**Myeloperoxidase (IU/mg protein)**	**D**_**1**_	15.66 ± 0.904c	17.34 ± 0.562b	20.65 ± 1.047a
	**D**_**2**_	16.09 ± 0.673c	13.53 ± 0.455d	13.28 ± 0.585d
	**D**_**3**_	16.08 ± 0.612c	12.42 ± 0.605e	10.85 ± 0.482f
**Nitric oxide (nmol/dL )**	**D**_**1**_	23.42 ± 1.35d	25.98 ± 0.84b	27.13 ± 1.38a
	**D**_**2**_	23.47 ± 0.98c	21.58 ± 0.73d	21.22 ± 0.93d
	**D**_**3**_	24.79 ± 0.94bc	21.21 ± 1.03d	17.43 ± 0.77e

Means sharing same letters in a column/row do not differ significantly at P < 0.05.

D_1_ = control diet; D_2_ *= N. sativa* seed fixed oil; D_3_ = *N. sativa* seed essential oil.

### Correlation matrix

The complications associated with diabetes mellitus are often associated with the production of free radicals and oxidative stress. The indices like antioxidant status and expression of various enzymes are important determinants. For this, correlation matrix was designed to check the interdependence of these attributes on each other (Table 5). It is obvious from the correlation coefficients that serum glucose level is positively associated with indices of immunopotentiating perspectives, i.e. xanthine oxidase and nitric oxide (P < 0.01). However, glucose is in negative association with insulin (P < 0.01), total antioxidant capacity (P < 0.05), and indices of antioxidant status, i.e. glutathione, α- and γ-tocopherols (P < 0.05). Furthermore, hepatic enzymes including glutathione peroxidase, glutathione reductase and glutathione transferase are in linear relationship with antioxidant status and glutathione contents (P < 0.05) while superoxide dismutase (SOD) was positively associated with this trait.

**Table 5:** Correlation matrix of some important parameters in diabetic rats

	**GSH**	**α-Toc**	**γ-Toc**	**SOD**	**CAT**	**GLPx**	**GR**	**GST**	**MPO**	**XO**	**NO**
**GSH**	1.00										
**α-Toc**	0.96**	1.00									
**γ-Toc**	0.85**	0.84**	1.00								
**SOD**	-0.87**	-0.84**	-0.77*	1.00							
**CAT**	0.83**	0.77*	0.92**	-0.79**	1.00						
**GLPx**	0.74*	0.72*	0.90**	-0.63^s^	0.94**	1.00					
**GR**	0.73*	0.72*	0.85**	-0.57^s^	0.88**	0.95**	1.00				
**GST**	0.78*	0.77*	0.94**	-0.69^ns^	0.95**	0.99**	0.94**	1.00			
**MPO**	-0.90**	-0.86**	-0.97**	0.79**	-0.94**	-0.92**	-0.86**	-0.95**	1.00		
**XO**	-0.90**	-0.87**	-0.87**	0.84**	-0.91**	-0.83**	-0.88**	-0.85**	0.88**	1.00	
**NO**	-0.94**	-0.89**	-0.90**	0.93**	-0.88**	-0.78*	-0.70*	-0.83**	0.94**	0.87**	1.00

ns = non-significant; * = significant (p >0.05); ** = highly significant (p>0.01).

GSH = glutathione; α-Toc = α-Tocopherol; γ-Toc = γ-Tocopherol; SOD = superoxide dismutase; CAT = catalase; GPx = glutathione peroxidase; GR = glutathione reductase; GST = glutathione transferase; NO = nitric oxide; MPO = myeloperoxidase; XO = xanthine oxidase.

The correlation matrix clearly showed that most of these parameters linked with a chain of variables, but indices of antioxidant status (P < 0.05) are linked with MPO, xanthine oxidase, and nitric oxide (P < 0.01). The lower antioxidant status is linked with higher immune responses and *vice-versa* that in turn affect negatively the hepatic enzyme production.

## Discussion

Diabetes mellitus is associated with production of free radicals that impart some unfavorable changes in the body metabolism. Resultantly, these changes are important in determining the complications. Streptozotocin (STZ) has shown in some previous research studies to produce oxygen free radicals by stimulating H_2_O_2_ generation in pancreatic β-cells that can damage the β-cell membranes and results in depletion of intracellular nicotinamide adenine dinucleotide (NAD), thus leading to onset and progression of diabetes mellitus [23]. The increased free radical production results in diabetes complications including cardiac hypertrophy, myocardial infractions, liver and kidney damage, and indeed neurodegenerative disorders [24]. Most of these complications are due to excessive free radical production and immuno-suppression. In the present research, we attempted to address the complications associated with diabetes mellitus with special reference to the free radical production using *N. Sativa* fixed and essential oils. In this research, we induced diabetes mellitus in Sprague Dawley rats using streptozotocin (STZ) and assessed the effectiveness of *N. sativa* fixed and essential oils in 8 weeks trails in reducing oxidative damage, improving antioxidant potential, modulation of hepatic enzymes, and boosting immunity. The results of the present exploration indicated that production of free radicals increased. STZ results in depletion of antioxidant system in both blood and tissues and promotes the generation of free radicals [25]. *N. sativa* essential oil owing to its antioxidant potential is useful in controlling the diabetic complications in experimental diabetic rats [26]. Glutathione provides protection in oxidative injury by participating in the cellular defense system against oxidative damage. In our experiments, glutathione contents decreased by 25.72% in control group while increased by 3.86 and 20.83% in *N. sativa* fixed and essential oils groups, respectively. The enhanced activities of glutathione were negatively associated with blood glucose showing that improved antioxidant defense system protects body from deleterious effects of diabetes mellitus [27]. Oxidative stress enhanced the production of highly reactive oxygen radicals that are toxic to cells, particularly the cell membrane in which these radicals interact with the lipid bi-layer and produce lipid peroxides. However, endogenous antioxidant enzymes (e.g. SOD, CAT, and GSH-Px) are responsible for the detoxification of deleterious oxygen radicals [28]. The Hepatic SOD activity was significantly higher in STZ-induced diabetic rats compared to the control rats. Similar changes were also recorded for CAT activity in diabetes groups, whereas GSH-Px activity was markedly decreased in diabetic rats compared to the normal rats. Recently, Huang and coworkers [24] also explicated that oxidative stress results in decreased activities of hepatic enzymes in diabetes mellitus; subsequently yielding deleterious effects on body metabolism and homeostasis. The hyperglycemia results in decreased activities of liver enzymes as reported by Lapshina *et al*. [29]. The findings were in agreement with present results as activities of hepatic enzymes were decreased in control group [26]. Use of *N. sativa* fixed and essential oils resulted in marked increase in the activities of liver enzymes. These results agree with data reported in literature [12,30].

Many researchers designed studies to elucidate the role of *N. sativa* fixed and essential oils for management of diabetes and presented various hypotheses for its hypoglycemic perspectives. In this regard, Kanter *et al.*[11] observed insulinotropic and antidiabetic effects of *N. sativa* and thymoquinone at a dose of 400 and 50 mg/kg body weight/day, respectively. Earlier, Meral *et al*. [30] observed lower glucose (194.41 mg/dl) in diabetic rats treated with *N. sativa* as compared to control (340.43 mg/dl). Subsequently, Kanter, [31] also suggested that *N. sativa* essential oil at a dose of 0.2 ml/kg/day intraperitoneously exerts therapeutic effect in diabetes by decreasing oxidative stress and preserving pancreatic β-cell integrity.

Nitric oxide is an indicator of reactive nitrogen species. In the present study, *N. sativa* fixed and essential oils modulated this parameter positively. However, its expression was in negative association with some hepatic enzymes. Previously, El-Mahmoudy *et al.*[32] found that *N. sativa* fixed oil suppressed the nitric oxide production. In this regard, the anti-inflammatory potentials of thymoquinone and nigellone, components of *N. sativa* essential oil, are of considerable importance as they act immune boosters. In earlier study by the same authors, *N. sativa* suppressed KBrO_3_ mediated renal oxidative stress, and multiple organ toxicity [27]. Overall, results of present study further supported the traditional use of *N. sativa* and its derived products as a treatment for hyperglycemia and related abnormalities. Moreover, *N. sativa* fixed and essential oils significantly ameliorate free radicals and improve antioxidant capacity thus reducing the risk of diabetic complications.

## Conclusions

Results of present study supported the traditional use of *N. sativa* fixed and essential oils significantly ameliorate free radicals and improve antioxidant status through modulation of hepatic enzyme expression and boosting immunity in animal models.

### Ethic statement

The present research was conducted following the instructions of “Animal Care Committee, NIFSAT-Faisalabad Pakistan” and research plan was submitted to National Institute of Health (NIH), Islamabad for further approval of research. They modified and approved the study after deliberate discussion and provided infectious free Sprague dawley rats as per requirements of modified proposed research. Thus, undertaken study does not violate the ethical and moral values for designing studies that would be used for planning further experimentation in human.

## Competing interests

The authors declare that they have no competing interests.

## Authors’ contributions

MTS, RK, MSB, SZI and designed and carried out the experimental work. SA, MZUH and ANA analyzed the statistical data and interpretation of results. LA and VDF drafted and critically evaluated the manuscript. All authors read and approved the final manuscript.

## Pre-publication history

The pre-publication history for this paper can be accessed here:

http://www.biomedcentral.com/1472-6882/14/193/prepub
